# Identification of Human Brain Proteins for Bitter-Sweet Taste Perception: A Joint Proteome-Wide and Transcriptome-Wide Association Study

**DOI:** 10.3390/nu14102177

**Published:** 2022-05-23

**Authors:** Wenming Wei, Bolun Cheng, Dan He, Yijing Zhao, Xiaoyue Qin, Qingqing Cai, Na Zhang, Xiaoge Chu, Sirong Shi, Feng Zhang

**Affiliations:** Key Laboratory of Trace Elements and Endemic Diseases, Collaborative Innovation Center of Endemic Disease and Health Promotion for Silk Road Region, School of Public Health, Health Science Center, Xi’an Jiaotong University, Xi’an 710061, China; wenmingwei@stu.xjtu.edu.cn (W.W.); cblbs1@stu.xjtu.edu.cn (B.C.); hedan1027367561@stu.xjtu.edu.cn (D.H.); zhaoyijing@stu.xjtu.edu.cn (Y.Z.); qinxiaoyue@stu.xjtu.edu.cn (X.Q.); caiqingqing@stu.xjtu.edu.cn (Q.C.); zhangna2021@stu.xjtu.edu.cn (N.Z.); 3121315054@stu.xjtu.edu.cn (X.C.); Shisirong@stu.xjtu.edu.cn (S.S.)

**Keywords:** bitterness and sweetness, taste perception, human brain proteins, brain development

## Abstract

Objective: Bitter or sweet beverage perception is associated with alterations in brain structure and function. Our aim is to analyze the genetic association between bitter or sweet beverage perception and human brain proteins. Materials and methods: In our study, 8356 and 11,518 proteins were first collected from two reference datasets of human brain proteomes, the ROS/MAP and Banner. The bitter or sweet beverage perception-related proteome-wide association studies (PWAS) were then conducted by integrating recent genome-wide association study (GWAS) data (*n* = 422,300) of taste perception with human brain proteomes. The human brain gene expression profiles were collected from two reference datasets, including the brain RNA-seq (CBR) and brain RNA-seq splicing (CBRS). The taste perception-related transcriptome-wide association studies (TWAS) were finally performed by integrating the same GWAS data with human brain gene expression profiles to validate the PWAS findings. Results: In PWAS, four statistically significant proteins were identified using the ROS/MAP and then replicated using the Banner reference dataset (all permutated *p <* 0.05), including *ABCG2* for total bitter beverages and tea, *CPNE1* for total bitter beverage, *ACTR1B* for artificially sweetened beverages, *FLOT2* for alcoholic bitter beverages and total sweet beverages. In TWAS analysis, six statistically significant genes were detected by CBR and confirmed by the CBRS reference dataset (all permutated *p <* 0.05), including *PIGG* for total bitter beverages and non-alcoholic bitter beverages, *C3orf18* for total bitter beverages, *ZSWIM7* for non-alcoholic bitter beverages, *PEX7* for coffee, *PKP4* for tea and *RPLP2* for grape juice. Further comparison of the PWAS and TWAS found three common statistically significant proteins/genes identified from the Banner and CBR reference datasets, including *THBS4* for total bitter beverages, *CA4* for non-alcoholic bitter beverages, *LIAS* for non-grape juices. Conclusions: Our results support the potential effect of bitter or sweet beverage perception on brain function and identify several candidate brain proteins for bitter or sweet beverage perception.

## 1. Introduction

Taste perception and preference are determinants for food and beverage selection and consumption, which in turn affect body weight and health, and even cause chronic diseases [1]. Although the overall taste of a food is generally thought of as a gestalt that contains information about taste, smell and body sensation, taste is the ultimate determinant of identifying potential foods [1]. It is human nature to prefer sweetness over bitterness for certain tastes [2], but not all bitterness is unpleasant [3]. In certain foods, a limited degree of bitterness is expected and enjoyed, which helps balance the flavor of beverages and foods [3]. Accepting sweetness as a signal for calories and rejecting strong bitterness as a warning for toxins is a human brainstem reflex that occurs in the fetus and changes throughout life, but it will never be erased by experience [1]. Taste perception and preference are genetic determinants of beverage choices and consumption [4].

Basic taste falls into five categories (sweet, salty, sour, bitter, and umami), but the most common beverage flavors are predominantly bitter (e.g., coffee, tea, beer, red wine, liquor, grape juice) and sweet (e.g., sugar-sweetened beverages, artificially sweetened beverages) [5]. Previous studies have indicated that bitter or sweet beverage perception was associated with alterations in brain structure and function. Several researchers have found that increased sugar consumption may affect neural pathways implicated in hypothalamic networks and frontocortical-limbic networks [6]. Low-calorie sweeteners, such as sucralose, aspartame, and ACEK could induce hypothalamic endoplasmic reticulum stress [7]. Epidemiological studies have shown that moderate consumption of alcohol, such as wine and beer, was beneficial to cognitive function [8]. The main bitter components of beer, iso-alpha-acids (IAAs), enhance hippocampus-dependent memory and prefrontal cortex-associated cognitive function [9]. However, most current studies have been done by tracking the metabolism of beverage ingredients in the brain, however, there is limited research on the relationships between taste perception and human brain proteins.

Genome-wide association studies (GWAS) have been conducted to identify genetic variants for taste perception. For example, Victor and his colleagues performed a GWAS on 370,000 participants of European descent, studied their self-reported consumption of bitter and sweet beverages, and identified 17 relevant genetic loci [5]. While GWAS has a powerful ability in exploring complex diseases/traits associated with genetic variants, how these variants affect target traits is rarely ascertainable. Several reasons cause the limitation of GWAS. First, the genetic variants identified by GWAS usually have small phenotypic effect sizes, and hundreds of identified genetic variants can only explain a small proportion of estimated heritability. Second, most GWAS-identified variants are located in non-coding regions (such as intergenic or intronic regions) involved in the regulation of gene expression, which limits the clarification of genetic mechanisms of diseases/traits. Furthermore, GWAS ignores the relationship among genetic variants, DNA functional elements (e.g., gene expression/protein levels) and complex traits or diseases [10]. Accordingly, interpreting the associated variants and loci rather than focusing on SNPs is vital to understanding how genetic variation contributes to taste perception.

To compensate for the shortcomings of GWAS, some powerful approaches have been developed, including the proteome-wide association study (PWAS) and transcriptome-wide association study (TWAS), which leverage reference panels to discover gene-trait associations from GWAS datasets. PWAS is a newly developed protein-centric method for identifying protein-coding genes associated with studied phenotypes. The method considers the effect of genetic variants on gene function and ignores their abundance. PWAS captures any variant that affects the coding regions of genes and then assigns each protein-coding gene functional affecting scores [11]. Different from PWAS analysis, TWAS is developed to identify genes whose genetically-regulated expression is associated with some risks of complex diseases or traits. Furthermore, TWAS uses external expression reference panels, such as expression quantitative trait loci (eQTL) cohorts [10] to estimate the association of each gene to disease. TWAS was conducted for many traits and tissues [12]. Both PWAS and TWAS analysis are novel and powerful tools for genetic association studies and can be utilized to prioritize candidate causal genes and obtain more concrete and interpretable discoveries.

In this study, we explore the GWAS results at two levels, the protein level and gene expression level. Firstly, we performed PWAS analysis by integrating GWAS results with human brain proteomes. Then, we performed TWAS analysis to validate the results by integrating GWAS results with the cis-genetic component of gene expression. We aim to gain a better understanding of the genetic mechanisms underlying bitter-sweet taste perception and analyze the genetic association between bitter or sweet beverage perception and human brain proteins.

## 2. Materials and Methods

### 2.1. GWAS Dataset of Bitter and Sweet Beverage

The GWAS summary data of beverage consumption was derived from a recently published study [5], which consists of 422,300 participants of European descent from UK Biobank. Diet data were collected using a 24 h recall questionnaire (Oxford WebQ (Bette Liu, Oxford, UK)) from a subset of participants in the UK biobank. Two phenotypes were defined, and each consists of several sub-phenotypes. Total bitter beverages phenotype includes coffee, tea, grape juice and alcoholic bitter beverages (beer/cider, red wine and liquor), while the total sweet beverages phenotype includes sugar-sweetened beverages, artificially sweetened beverages, non-grape juices, hot chocolate and flavored milk. Genotyping was performed using Affymetrix UK BiLEVE Axiom (Affymetrix Research Services Laboratory, Santa Clara, CA, USA) and Affymetrix UK Biobank Axiom^®^ (Affymetrix Research Services Laboratory, Santa Clara, CA, USA) arrays. Genotype imputation was performed using the Haplotype Reference Consortium (HRC) v1.1 and UK10K reference panels by the Wellcome Trust Centre for Human Genetics and the University of Oxford. Variants were removed with sample outliers based on heterozygosity and missingness if the kinship coefficient was >0.0442, the minor allele frequency was <0.001 or the low imputation quality score was ≤0.3. Detailed information on genotyping, imputation, quality control and statistical analysis can be found in the published study [5].

### 2.2. PWAS Analysis of Bitter and Sweet Beverage

Following the standard pipeline of the FUSION software [13], PWAS analysis was performed by integrating the GWAS results with a discovery and a confirmation brain proteome reference dataset. Briefly, the two human brain proteome reference datasets were profiled from the human dorsolateral prefrontal cortex (dlPFC). The discovery brain proteome reference dataset [14] was derived from the Religious Orders Study and Rush Memory and Aging Project (ROS/MAP) cohorts, including 391 participants from two longitudinal clinical-pathologic cohort studies of aging and Alzheimer’s disease. After quality control, 8356 proteins of 391 subjects were included in our analysis. The confirmation brain proteome reference dataset [15] was derived from the Banner Sun Health Research Institute (Banner), recruiting 198 participants of European descent. After quality control, 152 subjects with 11,518 proteins were quantified in the confirmation PWAS. Our analysis used 1,190,321 HapMap SNPs from 489 individuals of European descent from the 1000 Genomes Project, which was commonly referred to as the linkage disequilibrium reference panel in FUSION. In our research, 2000 permutations were implemented to control the potential impact of multiple testing on our PWAS results. The proteins with a permutated *p*-value < 0.05 were considered as significant.

### 2.3. TWAS Analysis of Bitter and Sweet Beverage

The TWAS analysis was performed by the FUSION software (Version 1 February 2022) (http://gusevlab.org/projects/fusion/ (accessed on 1 May 2021) (Alexander Gusev, Boston, MA, USA)) [13]. Using the pre-computed gene expression weights of different tissues together with GWAS summary data, FUSION is capable to estimate the associations of each gene with target diseases in different tissues. In our study, TWAS analysis was performed based on the GWAS summary statistics as well as the gene expression weights of dlPFC, anterior cingulate cortex, frontal cortex, amygdala, cerebellum, and hippocampus from FUSION [13]. Briefly, the gene expression weights were calculated using the prediction models of FUSION and combined with the GWAS results to impute association statistics between gene expression levels and bitter-sweet taste perception. The Bayesian sparse linear mixed model (BSLMM) was utilized to compute the SNP-expression weights in the 1-Mb cis loci of the gene for a given gene [16]. The association test statistics between the predicted gene expression and target trait were calculated as ZTWAS = W’Z/(W’SW)1/2 [13]. Z denotes the scores of bitter-sweet taste perception, while W denotes the weights. S denotes the SNP-correlation covariance matrix. We accounted for linkage disequilibrium (LD) among SNPs and viewed the imputed gene expression data as a linear model of genotypes with weights. Similar to PWAS, 2000 permutations were implemented to control the potential impact of multiple testing on our TWAS results. The genes with a permutated *p*-value < 0.05 were considered as significant. More details on adjusting for confounding factors are shown in (Materials and Methods (Supplementary Material).

We focused on two transcriptome datasets from dlPFC for mutual validation and further confirmation of PWAS results: brain RNA-seq (CBR) and brain RNA-seq splicing (CBRS), in which all cis-variants of gene expression are heritable [13]. We also validated the PWAS results utilizing five other transcriptomic datasets to explore gustatory-related gene expression in other brain regions, including the anterior cingulate cortex, frontal cortex, amygdala, cerebellum, and hippocampus.

### 2.4. Brain-Related Phenotype Analysis

Candidate genes and proteins identified by PWAS and TWAS were searched for in the IEU Open GWAS project website to match the traits related to brain function and cranial nerves (https://gwas.mrcieu.ac.uk/ (accessed on 10 May 2021)).

## 3. Results

### 3.1. PWAS Results of Bitter and Sweet Beverage

In PWAS, four statistically significant proteins were identified in discovery (ROS/MAP) and confirmation (Banner) reference datasets. For bitter beverage consumption, *ABCG2* was associated with total bitter beverages (*P_PWAS-ROS/MAP_* = 2.99 × 10^−3^, *P_PWAS-Banner_* = 3.05 × 10^−3^) and tea (*P_PWAS-ROS/MAP_* = 7.65 × 10^−3^, *P _PWAS-Banner_* = 8.79 × 10^−3^). *CPNE1* was associated with total bitter beverage (*P_PWAS-ROS/MAP_* = 4.96 × 10^−3^, *P_PWAS-Banner_* = 1.34 × 10^−3^). For sweet beverage consumption, *ACTR1B* was associated with artificially sweetened beverages (*P_PWAS-ROS/MAP_* = 9.62 × 10^−3^, *P_PWAS-Banner_* = 1.59 × 10^−2^). In addition, *FLOT2* was associated with alcoholic bitter beverages (*P_PWAS-ROS/MAP_* = 2.68 × 10^−2^, *P_PWAS-Banner_* = 1.76 × 10^−5^) and total sweet beverages (*P_PWAS-ROS/MAP_* = 5.04 × 10^−3^, *P_PWAS-Banner_* = 4.04 × 10^−7^).

### 3.2. TWAS Results of Bitter and Sweet Beverage

In TWAS, six statistically significant genes were identified in CBR and CBRS reference datasets. For bitter beverage consumption, *PIGG* was associated with total bitter beverages (*P_TWAS-CBR_* = 3.97 × 10^−3^, *P_TWAS-CBRS_* = 4.51 × 10^−3^) and non-alcoholic bitter beverages (*P_TWAS-CBR_* = 2.06 × 10^−3^, *P_TWAS-CBRS_* = 2.35 × 10^−3^). *C3orf18* was associated with total bitter beverages (*P_TWAS-CBR_* = 6.12 × 10^−5^, *P_TWAS-CBRS_* = 2.17 × 10^−3^). *ZSWIM7* was associated with non-alcoholic bitter beverages (*P_TWAS-CBR_* = 4.62 × 10^−2^, *P_TWAS-CBRS_* = 4.39 × 10^−2^). *PEX7* was associated with coffee (*P_TWAS-CBR_* = 3.41 × 10^−3^, *P_TWAS-CBRS_* = 4.43 × 10^−3^). *PKP4* and *RPLP2* were associated with tea (*P_TWAS-CBR_* = 7.65 × 10^−3^, *P_TWAS-CBRS_* = 4.81 × 10^−3^) and grape juice (*P_TWAS-CBR_* = 7.67 × 10^−3^, *P_TWAS-CBRS_* = 7.22 × 10^−3^), separately. For sweet beverage consumption, we did not find consistent genes in these two datasets. The statistically significant proteins/genes identified in the PWAS analysis and the gene expressions in the TWAS analysis are shown in Table 1 and Supplementary Figures S1–S10.

We found three common proteins/genes detected by PWAS and TWAS analyses. *THBS4* was associated with total bitter beverages (*P_PWAS-Banner_* = 7.27 × 10^−3^, *P_TWAS-CBR_* = 1.14 × 10^−4^). *CA4* was associated with non-alcoholic bitter beverages (*P_PWAS-Banner_* = 2.32 × 10^−2^, *P_TWAS-CBR_* = 3.75 × 10^−2^). *LIAS* was associated with non-grape juices (*P_PWAS-Banner_* = 7.57 × 10^−3^, *P_TWAS-CBR_* = 8.28 × 10^−4^). 

### 3.3. Brain-Related Phenotype Analysis

By inputting four candidate proteins from PWAS and six candidate genes from TWAS into the IEU Open GWAS project website, we matched each of them with traits related to brain function and cranial nerves, such as *ABCG2* for narcolepsy (*p* = 7.90 × 10^−5^), for volume Left-Cerebellum-Cortex (*p* = 5.37 × 10^−5^) and for volume Right-Thalamus-Proper (*p* = 4.79 × 10^−5^), *CPNE1* for cognitive performance (*p* = 1.57 × 10^−4^), for intelligence (*p* = 1.10 × 10^−4^) and for mood swings (*p* = 2.70 × 10^−4^). Detailed results of brain-related phenotype analysis are shown in Table 2.

### 3.4. Exploration of Other Brain Regions

*CPNE1* was associated with total bitter beverage (*P_PWAS-ROS/MAP_* = 4.96 × 10^−3^, *P _PWAS-Banner_* = 1.34 × 10^−3^) in the frontal cortex (*P_TWAS_* = 1.6 × 10^−12^), amygdala (*P_TWAS_* = 4.61 × 10^−8^), anterior cingulate cortex (*P_TWAS_* = 1.02 × 10^−7^), and hippocampus (*P_TWAS_* = 7.2 × 10^−10^). *ACTR1B* was associated with artificially sweetened beverages (*P_PWAS-ROS/MAP_* = 9.62 × 10^−3^, *P_PWAS-Banner_* = 1.59 × 10^−2^) in the cerebellum (*P_TWAS_* = 1.91 × 10^−2^). The results are shown in Table S1 in the Supplementary Material.

## 4. Discussion

In this study, we performed the bitter or sweet beverage perception-related PWAS and TWAS to extend the GWAS results to protein level and gene expression level in the brain. The results showed that four statistically significant proteins were identified in PWAS, and six statistically significant genes were identified in TWAS. Moreover, three genes were found to be differentially expressed in proteome-wide and transcriptome-wide levels, including *CA4*, *LIAS* and *THBS4*. 

For non-alcoholic bitter beverage consumption, the *CA4* gene was identified in PWAS and TWAS analyses. *CA4* encodes a membrane-associated enzyme called carbonic anhydrase IV, which is located on the luminal surface of cerebral capillaries and associated with the blood–brain barrier, and is also concentrated in layers III and VI in the cortex, hippocampus and thalamus [16]. CA4 appears to be the more important extracellular carbonic anhydrase (CA) in the hippocampus [17], involved in neuronal regulation [17]. In the brain, CA4 is responsible for the regulation of intracellular pH transients associated with neural discharge [18]. Changes in endogenous pH can affect neuronal function, and the influence depends on the size, speed, and spread of endogenous pH changes [17]. The most important factor controlling these variables is the buffering capacity of CA4 to the extracellular fluids [18]. In the taste system, expression of *CA4* was detected in sour-sensing presynaptic taste cells which provide the glycosylphosphatidylinositol (GPI) anchors that retain CA4 on the cell surface, enabling CA4 to play a key role in the cellular sensation of carbonation [17,19]. Type III cells produce secondary responses to sweet, umami, and bitter stimuli [20]. CA4 is utilized as a type III cell marker and located on the surface of type III cells, converting on-site CO_2_ to bicarbonate and protons which are thought to locally stimulate sour transduction pathways in these cells [21]. However, uncertainties remain as to whether the relationship between non-alcoholic bitter beverage consumption and CA4 in the brain is related to the above mechanism, and further research is needed. 

For total bitter beverages consumption, *THBS4* was identified as statistically significant in PWAS and TWAS analyses. The *THBS4* gene encodes a large extracellular-matrix glycoprotein, thrombospondin 4. THBS4 directly acts as a synaptogenic factor on neurons and may represent a rejuvenation factor that enhances synaptic connectivity by increasing dendritic arborization, synapse formation, and synaptic transmission [22]. Experiments revealed that increasing thrombospondin levels could enhance cortical plasticity changes in adults by contributing a higher density of synapses, a higher rate of synaptic turnover, or some combination of these factors [23]. In mice, THBS4 protein has previously been linked to neurodegeneration and has recently been identified as a rejuvenation factor [22]. Other hypotheses suggest that THBS4 may facilitate laminin clustering and thereby increase dendritic branching, or it binds to integrins to stabilize synapses, or it modulates Notch signaling to affect synaptic plasticity. At present, the precise function of THBS4 as a synaptogenic factor is unclear [22]. Down-regulation of *THBS4* promoted neuronal regeneration and played a beneficial role in the recovery of nerve function [24]. RNA sequencing experiments indicated that the expression of *THBS4* increased in the human prefrontal cortex during life [25]. In short, THBS4 is involved in the development of the central nervous system. However, the relationship between bitter beverage consumption and the mechanism of *THBS4* in the brain remains unclear, and further research is needed.

Bitter beverages include coffee, tea, grape juice, red wine, liquor and beer. Some research has shown that bitter beverage consumption was related to brain function. For example, several epidemiological studies have indicated that moderate consumption of alcoholic beverages, such as wine and beer, may benefit cognitive function [8] and lower the risk of dementia [9]. The bitter component of beer, iso-alpha-acids, could improve hippocampus-dependent memory through vagus nerve activation [8]. Coffee or tea consumption was negatively correlated with cognitive decline [5]. Similar to the above results, our study found that *FLOT2* may affect cognitive performance and *ZSWIM7* may affect depression. Coffee consumption appears to be beneficial for Parkinson’s disease [26], depression [27] and cognitive disorders [28,29]. For tea consumption, *ABCG2* was detected, which was associated with narcolepsy, a disorder related to changes in brain function. Deeper mechanisms linking bitterness to brain function still need to be explored.

For non-grape juices consumption, *LIAS* was identified as statistically significant in PWAS and TWAS analyses. *LIAS* is one of the candidate genes to synthesize lipoic acid (LA). Alpha-lipoic acid has a redox-active disulfide group and acts as a cofactor of the E2 subunit of pyruvate dehydrogenase in mitochondria [30]. LA plays an antioxidant role that can significantly reduce lipid peroxidation levels, recover the catalase activity and dopamine levels, and reduce oxidative stress [31,32]. As a powerful antioxidant factor, LA could stimulate nerves and regenerate nerve fibers [33]. Moreover, LA protects cultured hippocampal neurons from beta-amyloid peptides-induced neurotoxicity [34]. Beta-amyloid deposition induces cerebral inflammation, which plays a major role in the neurodegenerative pathology underlying Alzheimer’s disease [35]. LA increased insulin sensitivity and reduced the manifestations of depressive disorder [33]. Studies have shown that LA was beneficial for neurodegenerative diseases, such as Alzheimer’s disease [34,35], Parkinson’s disease [31] and Huntington’s disease [32,34,36]. Our results suggest that non-grape juices preference may have an excellent effect on the central nervous system through the molecular action of LA encoded by *LIAS*.

We detected that *FLOT* was associated with alcoholic bitter beverages and total sweet beverages. Indeed, studies have proven that the perception of sweetness and bitterness is partially intertwined. Sweet, amino acid, and bitter taste receptor cells use a common signaling pathway to produce the taste response [3]. Despite using different receptor systems, sweetness, bitterness and umami are all detected by type II cells via G protein-coupled receptors [37]. On taste buds, gustatory receptors detect the taste stimuli on the tongue and then transmit sensory information to the solitary tract and thalamus via taste nerves [38]. Then, the thalamic pathway continues to a region of the insula known as the primary gustatory area and then to the secondary gustatory area in the orbitofrontal cortex, which relays the information to the hypothalamus, amygdala, and other brain regions [6]. Whether these mechanisms are related to the genes we have discovered here, remains to be further verified.

One of the innovations of our research is the comprehensive analysis of mutual verification of PWAS and TWAS, and each method uses two datasets for discovery and verification. Briefly, we combined proteomics and transcriptomics with GWAS results to interpret and explore GWAS-associated signals at the gene expression level and protein level and look for candidate causal genes for taste perception. In addition, the large sample size of taste perception consumption increases the accuracy and effectiveness of our analysis. Our results contribute to understanding the biological mechanisms of bitterness or sweetness perception.

Previous studies have reported relationships between the consumption of certain flavored beverages and brain activity [6,7,8,9,39,40]. However, most studies focus on links between beverage ingredients and brain regions or neural pathways, while less explore the relationship between taste and genes. Though our study can provide some new ideas, there are still some issues that need to be noted. Although the sample size of the GWAS summary data in our research is large, the sample is mainly of European descent. Future well-powered multi-ethnic GWAS should be utilized to confirm these results. Additionally, there is evidence that different tastes share a common genetic predisposition, so our results can be generalized to three other types of taste. Finally, reference datasets from different sources could be a potential bias.

In conclusion, we conducted a systematic analysis of the relationship between human brain proteins and taste perception (bitter and sweet). We identified several brain proteins which were associated with the consumption of bitter or sweet beverages, indicating the potential effect of bitter or sweet beverages preference on brain development.

## Figures and Tables

**Table 1:** Significant proteins or genes identified by PWAS and TWAS analysis for beverage consumption.

Beverage Type		Proteins/Genes			Chromosome	Permutation *p* Value	
		Symbol	EnsemblID	Name			
**PWAS**	Bitter beverages					**ROS/MAP**	**Banner**
	Total bitter beverages	*ABCG2*	ENSG00000118777	ATP binding cassette subfamily G member 2	4	2.99 × 10^−3^	3.05 × 10^−3^
	Total bitter beverages	*CPNE1*	ENSG00000214078	copine 1	20	4.96 × 10^−3^	1.34 × 10^−3^
	Alcoholic bitter beverages	*FLOT2*	ENSG00000132589	flotillin 2	17	2.68 × 10^−2^	1.76 × 10^−5^
	Tea	*ABCG2*	ENSG00000118777	ATP binding cassette subfamily G member 2	4	7.65 × 10^−3^	8.79 × 10^−3^
	Sweet beverages						
	Total sweet beverages	*FLOT2*	ENSG00000132589	flotillin 2	17	5.04 × 10^−3^	4.04 × 10^−7^
	Artificially sweetened beverages	*ACTR1B*	ENSG00000115073	actin related protein 1B	2	9.62 × 10^−3^	1.59 × 10^−2^
**TWAS**	Bitter beverages					**CBR**	**CBRS**
	Total bitter beverages	*PIGG*	ENSG00000174227	phosphatidylinositol glycan anchor biosynthesis class G	4	3.97 × 10^−3^	4.51 × 10^−3^
	Total bitter beverages	*C3orf18*	ENSG00000088543	chromosome 3 open reading frame 18	3	6.12 × 10^−5^	2.17 × 10^−3^
	Alcoholic bitter beverages	*ZSWIM7*	ENSG00000214941	zinc finger SWIM-type containing 7	17	4.62 × 10^−2^	4.39 × 10^−2^
	Non-alcoholic bitter beverages	*PIGG*	ENSG00000174227	phosphatidylinositol glycan anchor biosynthesis class G	4	2.06 × 10^−3^	2.35 × 10^−3^
	Coffee	*PEX7*	ENSG00000112357	peroxisomal biogenesis factor 7	6	3.41 × 10^−3^	4.43 × 10^−3^
	Tea	*PKP4*	ENSG00000144283	plakophilin 4	2	7.65 × 10^−3^	4.81 × 10^−3^
	Grape juice	*RPLP2*	ENSG00000177600	ribosomal protein lateral stalk subunit P2	11	7.67 × 10^−3^	7.22 × 10^−3^

Note: PWAS, proteome-wide association study; TWAS, transcriptome-wide association study. ROS/MAP and Banner means human brain proteomes for PWAS analysis; CBR and CBRS means two datasets of human brain gene expressions for TWAS analysis. Overlapped genes/proteins identified by PWAS and TWAS.

**Table 2:** Brain-related phenotype of significant proteins or genes identified by PWAS and TWAS analysis.

Beverage Type		Proteins/Genes			Brain-Related Phenotype	*p* Value
		Symbol	EnsemblID	Name		
**PWAS**	Bitter beverages					
	Total bitter beverages	*ABCG2*	ENSG00000118777	ATP binding cassette subfamily G member 2	Narcolepsy	7.90 × 10^−5^
					Volume Left-Cerebellum-Cortex	5.37 × 10^−5^
					Volume Right-Thalamus-Proper	4.79 × 10^−5^
		*CPNE1*	ENSG00000214078	copine 1	Cognitive performance	1.57 × 10^−4^
					Intelligence	1.10 × 10^−4^
					Mood swings	2.70 × 10^−4^
	Alcoholic bitter beverages	*FLOT2*	ENSG00000132589	flotillin 2	Cognitive performance	8.13 × 10^−6^
					Neuroticism	3.35 × 10^−4^
					Tense/‘highly strung’	3.20 × 10^−4^
	Tea	*ABCG2*	ENSG00000118777	ATP binding cassette subfamily G member 2	Narcolepsy	7.90 × 10^−5^
					Volume Left-Cerebellum-Cortex	5.37 × 10^−5^
					Volume Right-Thalamus-Proper	4.79 × 10^−5^
	Sweet beverages					
	Total sweet beverages	*FLOT2*	ENSG00000132589	flotillin 2	Cognitive performance	8.13 × 10^−6^
					Neuroticism	3.35 × 10^−4^
					Tense/‘highly strung’	3.20 × 10^−4^
	Artificially sweetened beverages	*ACTR1B*	ENSG00000115073	actin related protein 1B	Bipolar disorder	1.40 × 10^−4^
					Schizophrenia	2.93 × 10^−4^
					Ever depressed for a whole week	2.84 × 10^−4^
**TWAS**	Bitter beverages					
	Total bitter beverages	*PIGG*	ENSG00000174227	phosphatidylinositol glycan anchor biosynthesis class G	Not found	/
		*C3orf18*	ENSG00000088543	chromosome 3 open reading frame 18	Intelligence	4.50 × 10^−4^
					Anxiety, nerves or generalized anxiety disorder	4.91 × 10^−4^
					Mood swings	7.50 × 10^−4^
	Alcoholic bitter beverages	*ZSWIM7*	ENSG00000214941	zinc finger SWIM-type containing 7	Depressed affect	8.96 × 10^−4^
					Parkinson’s disease	3.07 × 10^−7^
					Feeling miserable	2.50 × 10^−5^
	Non-alcoholic bitter beverages	*PIGG*	ENSG00000174227	phosphatidylinositol glycan anchor biosynthesis class G	Not found	/
	Coffee	*PEX7*	ENSG00000112357	peroxisomal biogenesis factor 7	Easily tired during worst period of anxiety	4.90 × 10^−4^
					IDP T1 FAST ROIs R heschl gyrus	5.75 × 10^−4^
	Tea	*PKP4*	ENSG00000144283	plakophilin 4	Manic/hyper symptoms	8.80 × 10^−4^
	Grape juice	*RPLP2*	ENSG00000177600	ribosomal protein lateral stalk subunit P2	DKTatlas lh paracentral area	4.17 × 10^−4^
					Manic/hyper symptoms	1.66 × 10^−4^
					IDP SWI T2star left thalamus	5.89 × 10^−4^

Note: PWAS, proteome-wide association study; TWAS, transcriptome-wide association study. ROS/MAP and Banner means human brain proteomes for PWAS analysis; CBR and CBRS means two datasets of human brain gene expressions for TWAS analysis. IDP, imaging derived phenotype; ROI, region of interest; DKT, Desikan–Killiany–Tourville cortical labeling protocol; SWI, susceptibility weighted imaging. FAST is a segmentation sentence in a brain image analysis software.

## Data Availability

The datasets generated during and/or analyzed during the current study are available from the corresponding authors upon reasonable request.

## Supplementary Materials

The following supporting information can be downloaded at: https://www.mdpi.com/article/10.3390/nu14102177/s1, Figure S1: Manhattan Plots of significant human brain proteins identified in PWAS and TWAS analysis for total bitter beverages; Figure S2: Manhattan Plots of significant human brain proteins identified in PWAS and TWAS analysis for non-alcoholic bitter beverages; Figure S3: Manhattan Plots of significant human brain proteins identified in PWAS and TWAS analysis for non-grape juices; Figure S4: Manhattan Plots of significant human brain proteins identified in PWAS and TWAS analysis for alcoholic bitter beverages; Figure S5: Manhattan Plots of significant human brain proteins identified in PWAS and TWAS analysis for coffee; Figure S6: Manhattan Plots of significant human brain proteins identified in PWAS and TWAS analysis for tea; Figure S7: Manhattan Plots of significant human brain proteins identified in PWAS and TWAS analysis for grape juice; Figure S8: Manhattan Plots of significant human brain proteins identified in PWAS and TWAS analysis for total sweet beverages; Figure S9: Manhattan Plots of significant human brain proteins identified in PWAS and TWAS analysis for sugar-sweetened beverages; Figure S10: Manhattan Plots of significant human brain proteins identified in PWAS and TWAS analysis for artificially sweetened beverages; Table S1: Exploration of other brain regions.
