# Supplementary material for: Identification of Human Brain Proteins for Bitter-Sweet Taste Perception: A Joint Proteome-Wide and Transcriptome-Wide Association Study
Source: Nutrients. 2022 May 23;14(10):2177. doi: 10.3390/nu14102177 (PMC9143225; doi:10.3390/nu14102177)
Supplement: Supplementary file 1 [file nutrients-14-02177-s001.zip › Materials and Methods.pdf]

Materials and Methods

For two brain proteome reference datasets(*1*), samples were randomized by age, sex, postmortem interval, cognitive diagnosis and pathologies into 50 batches before tandem mass tag labeling to minimize batch effects. To remove the effects of protein loading differences, each protein abundance was scaled with a sample-specific total protein abundance and log2-transformed the abundance. Next, poorly performing samples were identified and removed using iterative principal component analysis to remove samples with greater than four standard deviations from the mean of either the first or second principal component. Subsequently, regression was used to estimate and remove the effects of proteomic sequencing batch, mass spectrometry reporter quantification mode, sex, age at death, postmortem interval and the final clinical diagnosis of cognitive status from the proteomic profile.

For two gene expression reference datasets(*2*), Picard v.1.83 was used to convert BAM files to FASTQ format and STAR v.2.5.1b31 was used to align reads to the GRCh38 reference genome and compute gene counts for each sample. Genes were removed with <1 count per million in at least 50% of the samples and with missing gene length and percentage guanine–cytosine content. Next, outlier samples were removed. Then, regression was used to remove effects of batch, sex, postmortem interval, age at death, brain region and final diagnosis of cognitive status from the transcriptomic profiles before estimating mRNA weights.

For GWAS summary data(*3*), statistical models for phenotypes based on 24 h recall data were adjusted for age, sex, BMI, total energy, proportion of 24 h recalls self-reported as capturing ‘typical intake’ and top 20 principal components to account for population sub-structure. The same adjustment was applied to the three sub-phenotypes based on the touchscreen questionnaire with the exception of total energy and ‘typical intake’.

1. A. P. Wingo *et al.*, Integrating human brain proteomes with genome-wide association data implicates new proteins in Alzheimer's disease pathogenesis. *Nat Genet* **53**, 143-146 (2021).
2. A. Gusev *et al.*, Integrative approaches for large-scale transcriptome-wide association studies. *Nat Genet* **48**, 245-252 (2016).
3. V. W. Zhong *et al.*, A genome-wide association study of bitter and sweet beverage consumption. *Human Molecular Genetics* **28**, 2449-2457 (2019).
