# Supplementary material for: Identification of Human Brain Proteins for Bitter-Sweet Taste Perception: A Joint Proteome-Wide and Transcriptome-Wide Association Study
Source: Nutrients. 2022 May 23;14(10):2177. doi: 10.3390/nu14102177 (PMC9143225; doi:10.3390/nu14102177)
Supplement: Supplementary file 1 [file nutrients-14-02177-s001.zip › Supplementary Figure.pdf]

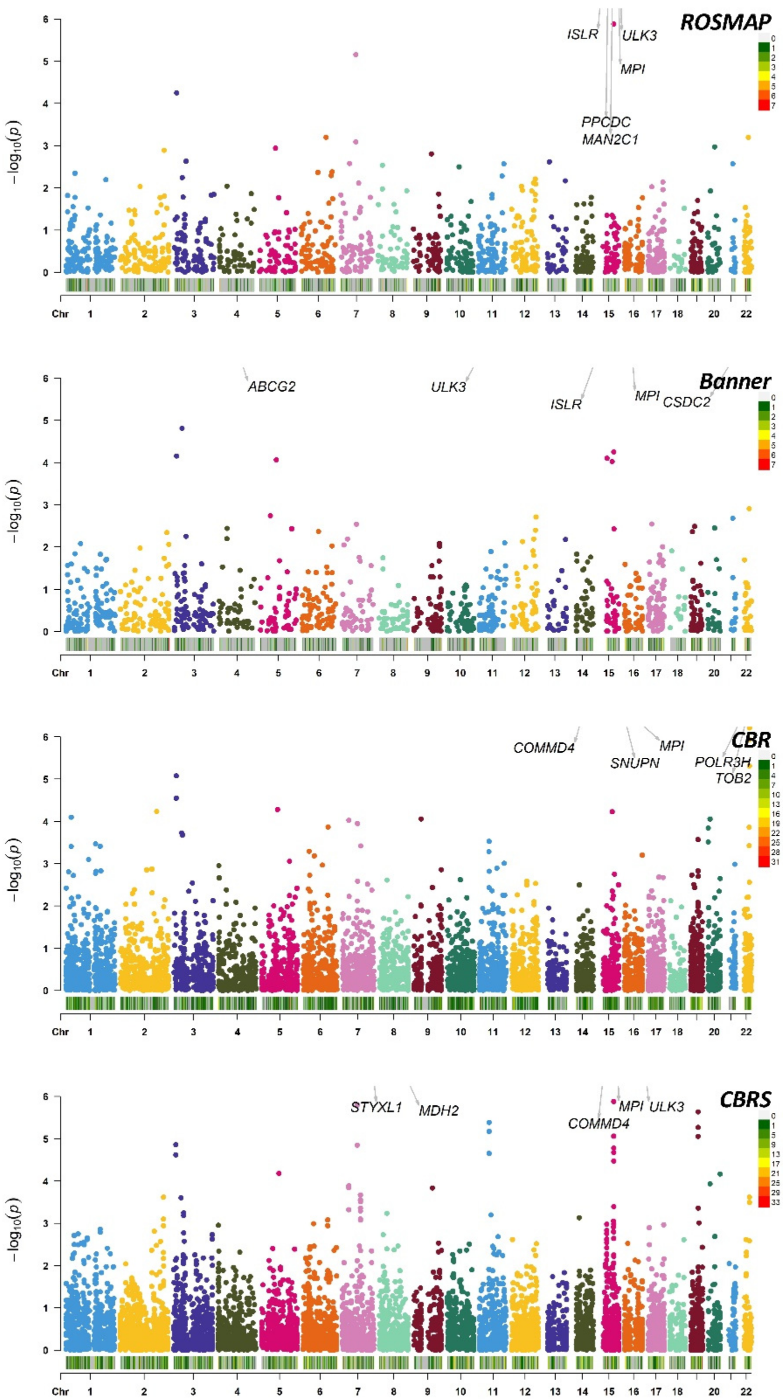

Figure S1. Manhattan Plots of significant human brain proteins identified in PWAS and TWAS analysis for total bitter beverages

Note: PWAS, proteome-wide association study; TWAS, transcriptome-wide association study. ROS/MAP and Banner means human brain proteomes for PWAS analysis; CBR and CBRS means two datasets of human brain gene expressions for TWAS analysis. Each point corresponds to a single test of association between a gene and phenotype, plotted according to genomic position on the x-axis and the strength of association ($-\log_{10}$ *P*-value) on the y-axis. The five most statistically significant genes/proteins resulting from the analysis of each cohort were mapped out.

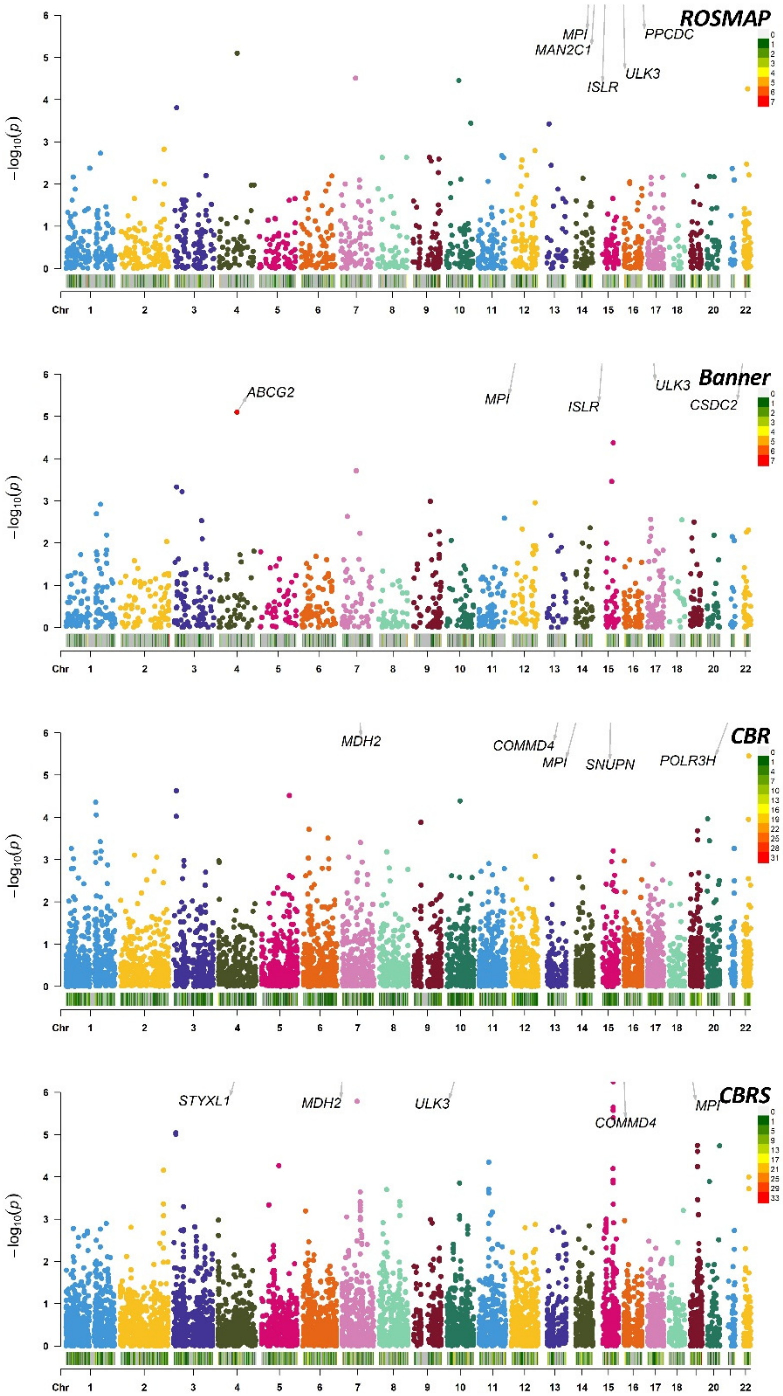

Figure S2. Manhattan Plots of significant human brain proteins identified in PWAS and TWAS analysis for non-alcoholic bitter beverages

Note: PWAS, proteome-wide association study; TWAS, transcriptome-wide association study. ROS/MAP and Banner means human brain proteomes for PWAS analysis; CBR and CBRS means two datasets of human brain gene expressions for TWAS analysis. Each point corresponds to a single test of association between a gene and phenotype, plotted according to genomic position on the x-axis and the strength of association ($-\log_{10}$ *P*-value) on the y-axis. The five most statistically significant genes/proteins resulting from the analysis of each cohort were mapped out.

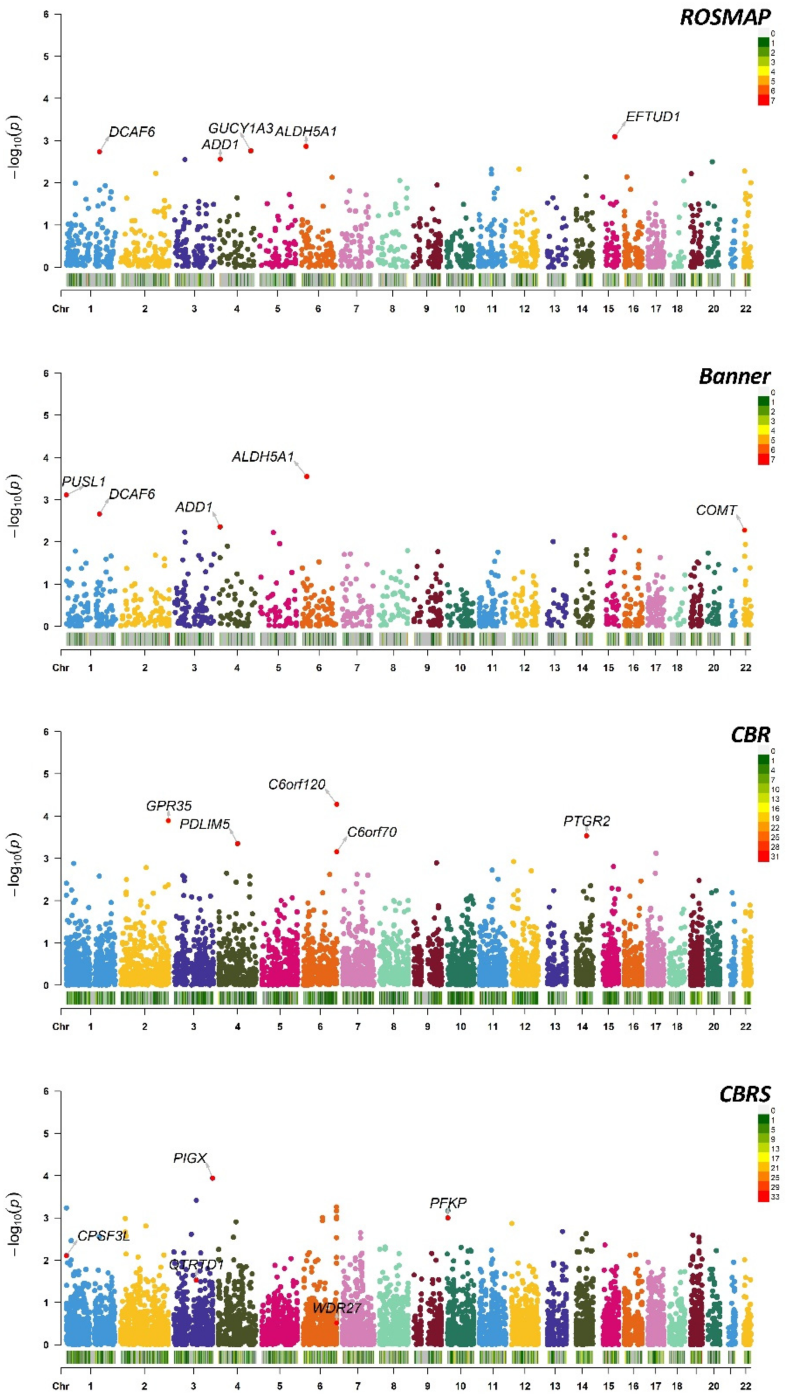

Figure S3. Manhattan Plots of significant human brain proteins identified in PWAS and TWAS analysis for non-grape juices

Note: PWAS, proteome-wide association study; TWAS, transcriptome-wide association study. ROS/MAP and Banner means human brain proteomes for PWAS analysis; CBR and CBRS means two datasets of human brain gene expressions for TWAS analysis. Each point corresponds to a single test of association between a gene and phenotype, plotted according to genomic position on the x-axis and the strength of association ($-\log_{10} P$-value) on the y-axis. The five most statistically significant genes/proteins resulting from the analysis of each cohort were mapped out.

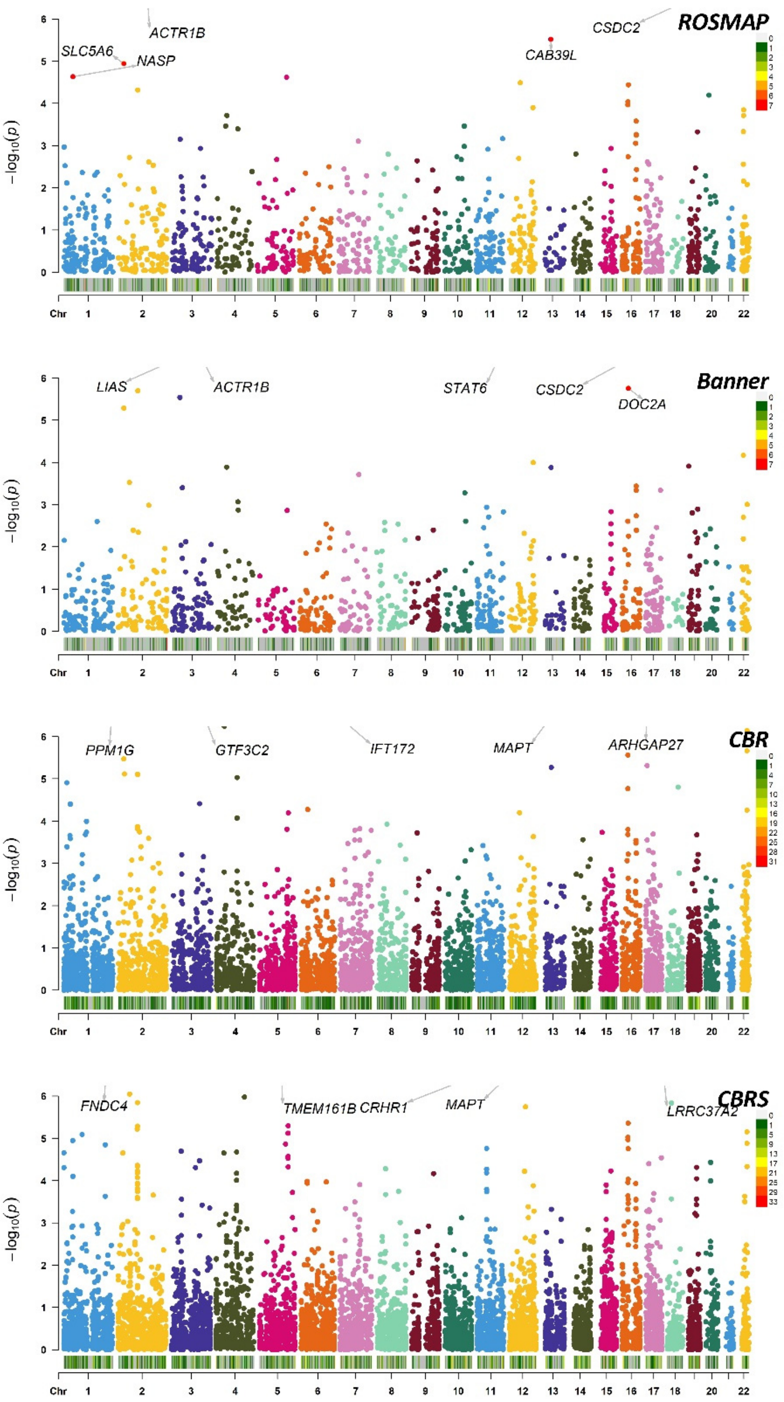

Figure S4. Manhattan Plots of significant human brain proteins identified in PWAS and TWAS analysis for alcoholic bitter beverages

Note: PWAS, proteome-wide association study; TWAS, transcriptome-wide association study. ROS/MAP and Banner means human brain proteomes for PWAS analysis; CBR and CBRS means two datasets of human brain gene expressions for TWAS analysis. Each point corresponds to a single test of association between a gene and phenotype, plotted according to genomic position on the x-axis and the strength of association (−log10 *P*-value) on the y-axis. The five most statistically significant genes/proteins resulting from the analysis of each cohort were mapped out.

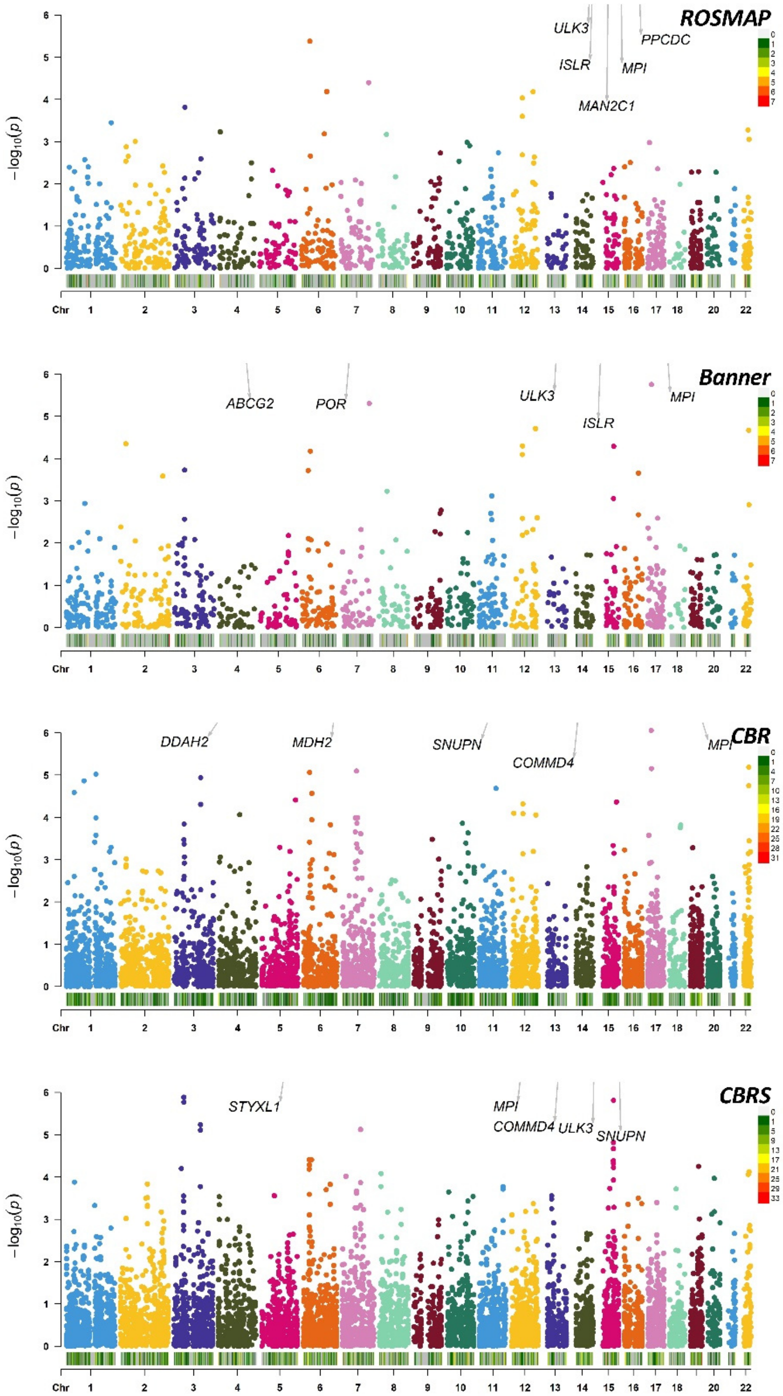

Figure S5. Manhattan Plots of significant human brain proteins identified in PWAS and TWAS analysis for coffee

Note: PWAS, proteome-wide association study; TWAS, transcriptome-wide association study. ROS/MAP and Banner means human brain proteomes for PWAS analysis; CBR and CBRS means two datasets of human brain gene expressions for TWAS analysis. Each point corresponds to a single test of association between a gene and phenotype, plotted according to genomic position on the x-axis and the strength of association ($-\log_{10}$ *P*-value) on the y-axis. The five most statistically significant genes/proteins resulting from the analysis of each cohort were mapped out.

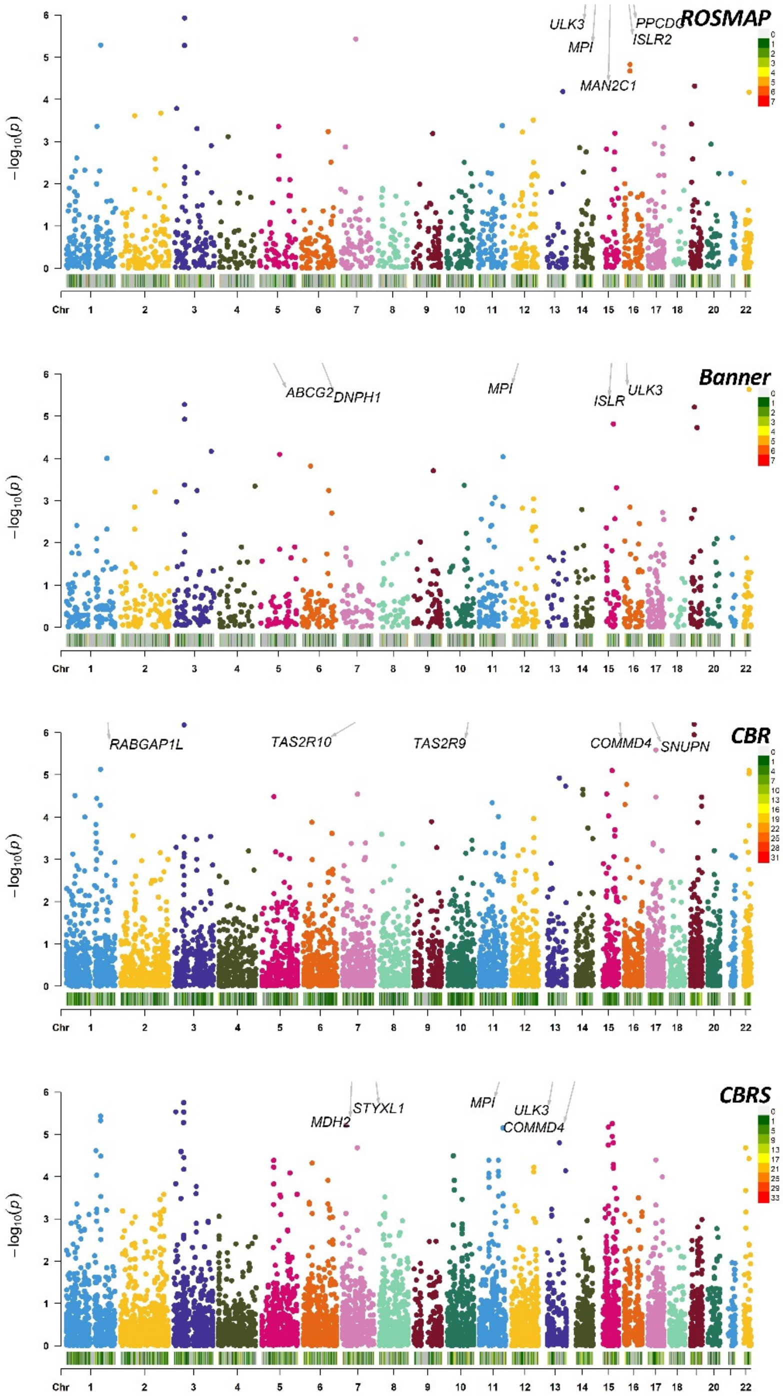

Figure S6. Manhattan Plots of significant human brain proteins identified in PWAS and TWAS analysis for tea

Note: PWAS, proteome-wide association study; TWAS, transcriptome-wide association study. ROS/MAP and Banner means human brain proteomes for PWAS analysis; CBR and CBRS means two datasets of human brain gene expressions for TWAS analysis. Each point corresponds to a single test of association between a gene and phenotype, plotted according to genomic position on the x-axis and the strength of association ($-\log_{10}$ *P*-value) on the y-axis. The five most statistically significant genes/proteins resulting from the analysis of each cohort were mapped out.

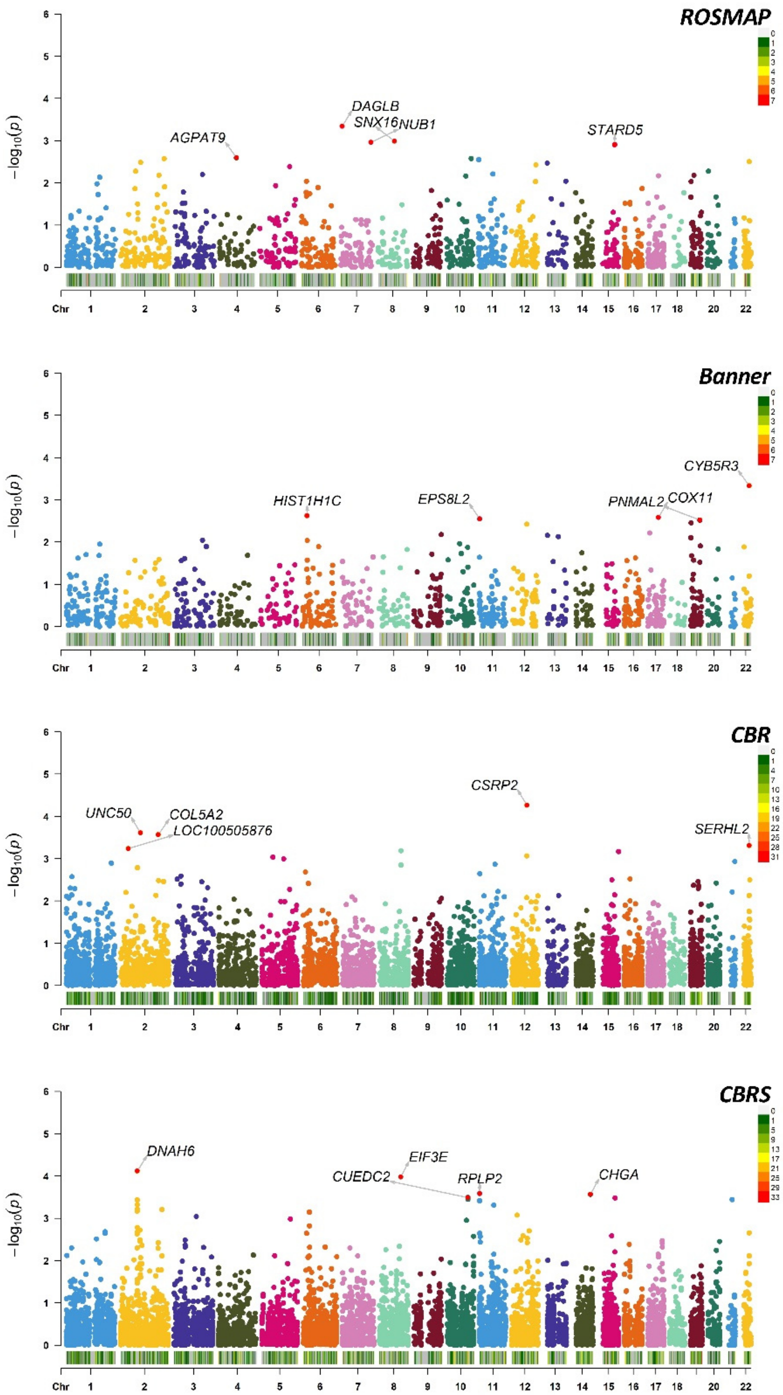

Figure S7. Manhattan Plots of significant human brain proteins identified in PWAS and TWAS analysis for grape juice

Note: PWAS, proteome-wide association study; TWAS, transcriptome-wide association study. ROS/MAP and Banner means human brain proteomes for PWAS analysis; CBR and CBRS means two datasets of human brain gene expressions for TWAS analysis. Each point corresponds to a single test of association between a gene and phenotype, plotted according to genomic position on the x-axis and the strength of association ($-\log_{10}$ *P*-value) on the y-axis. The five most statistically significant genes/proteins resulting from the analysis of each cohort were mapped out.

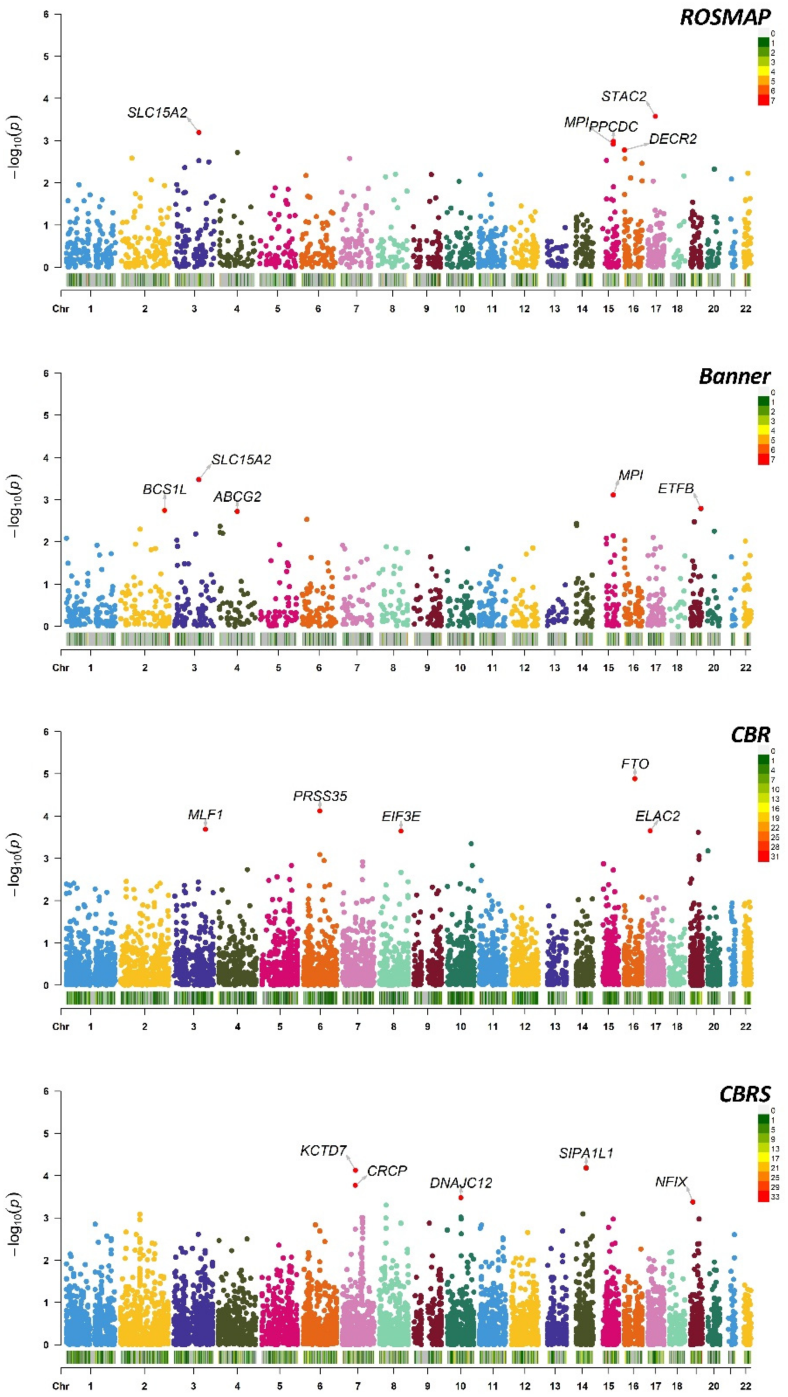

Figure S8. Manhattan Plots of significant human brain proteins identified in PWAS and TWAS analysis for total sweet beverages

Note: PWAS, proteome-wide association study; TWAS, transcriptome-wide association study. ROS/MAP and Banner means human brain proteomes for PWAS analysis; CBR and CBRS means two datasets of human brain gene expressions for TWAS analysis. Each point corresponds to a single test of association between a gene and phenotype, plotted according to genomic position on the x-axis and the strength of association ($-\log_{10}$ *P*-value) on the y-axis. The five most statistically significant genes/proteins resulting from the analysis of each cohort were mapped out.

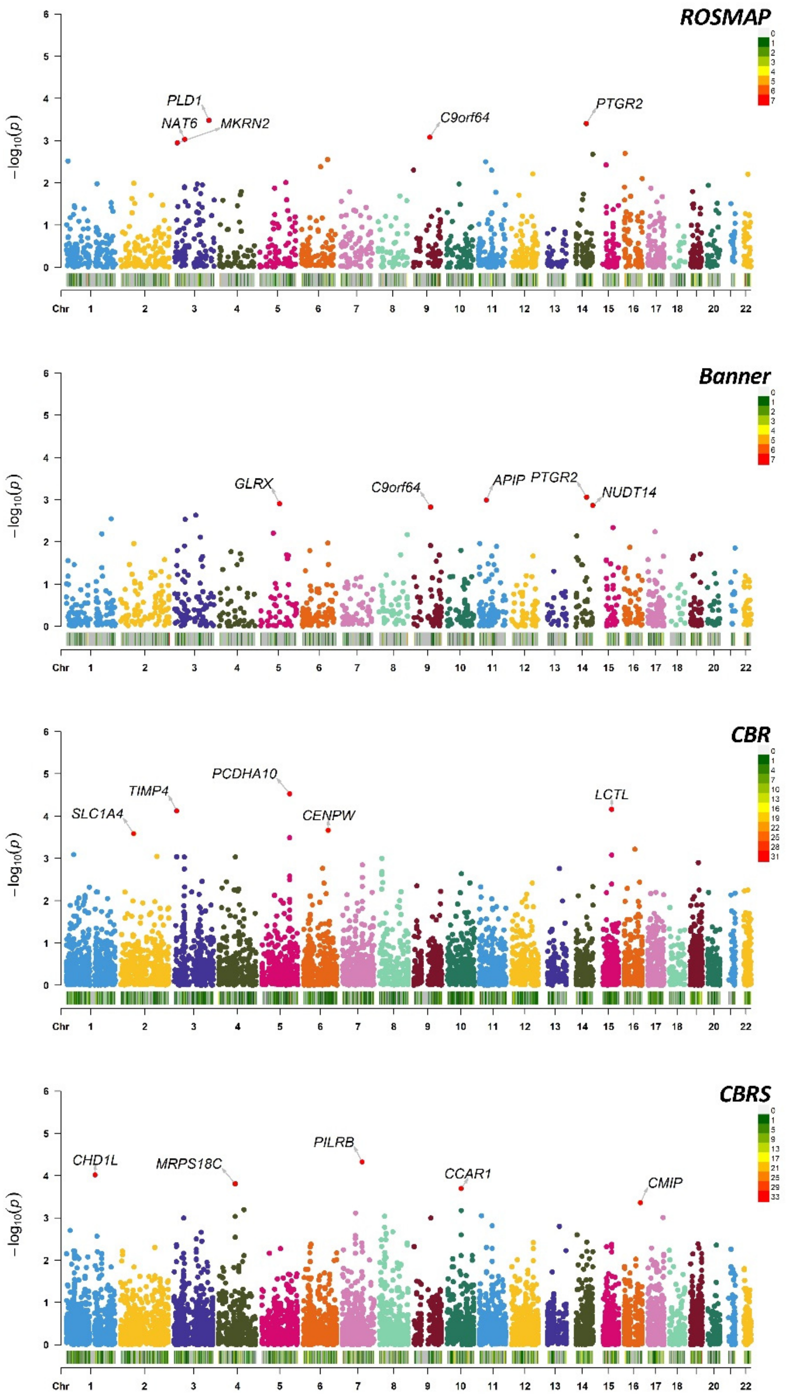

Figure S9. Manhattan Plots of significant human brain proteins identified in PWAS and TWAS analysis for sugar-sweetened beverages

Note: PWAS, proteome-wide association study; TWAS, transcriptome-wide association study. ROS/MAP and Banner means human brain proteomes for PWAS analysis; CBR and CBRS means two datasets of human brain gene expressions for TWAS analysis. Each point corresponds to a single test of association between a gene and phenotype, plotted according to genomic position on the x-axis and the strength of association ($-\log_{10}$ *P*-value) on the y-axis. The five most statistically significant genes/proteins resulting from the analysis of each cohort were mapped out.

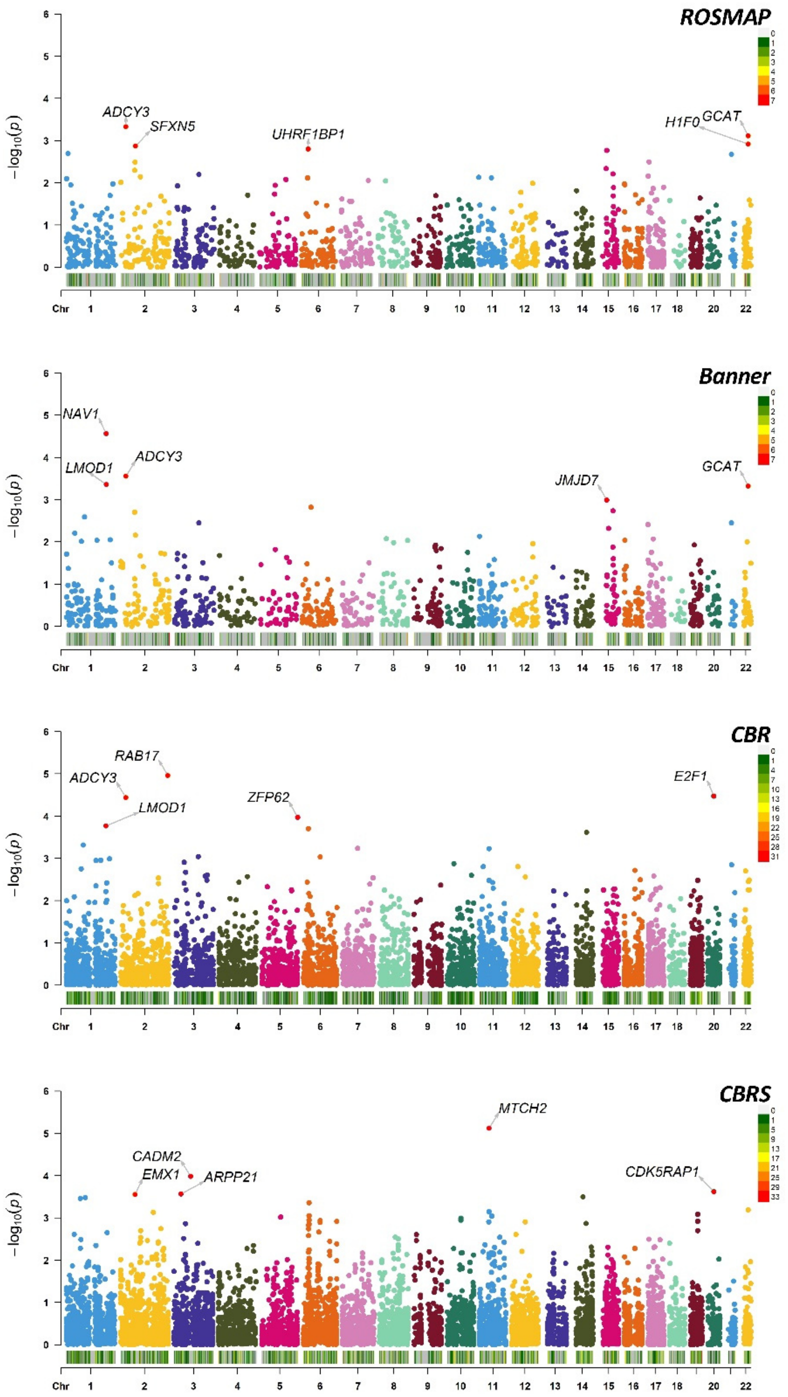

Figure S10. Manhattan Plots of significant human brain proteins identified in PWAS and TWAS analysis for artificially sweetened beverages

Note: PWAS, proteome-wide association study; TWAS, transcriptome-wide association study. ROS/MAP and Banner means human brain proteomes for PWAS analysis; CBR and CBRS means two datasets of human brain gene expressions for TWAS analysis. Each point corresponds to a single test of association between a gene and phenotype, plotted according to genomic position on the x-axis and the strength of association ($-\log_{10}$ *P*-value) on the y-axis. The five most statistically significant genes/proteins resulting from the analysis of each cohort were mapped out.
