# Supplementary material for: Identification of Human Brain Proteins for Bitter-Sweet Taste Perception: A Joint Proteome-Wide and Transcriptome-Wide Association Study
Source: Nutrients. 2022 May 23;14(10):2177. doi: 10.3390/nu14102177 (PMC9143225; doi:10.3390/nu14102177)
Supplement: Supplementary file 1 [file nutrients-14-02177-s001.zip › Supplementary material.pdf]

Table S1 Exploration of other brain regions

Beverage type	Genes	PWAS (Permutation *P* value)		TWAS (Permutation *P* value)				
		ROS/MAP	Banner	Frontal Cortex	Amygdala	Cerebellum	Anterior cingulate cortex	Hippocampus
Bitter beverages								
Total	*ABCG2*	2.99×10^{-3}	3.05×10^{-3}	/	/	/	/	/
Total	*CPNE1*	4.96×10^{-3}	1.34×10^{-3}	1.6×10^{-12}	4.61×10^{-08}	/	1.02×10^{-07}	7.2×10^{-10}
Alcoholic bitter beverages	*FLOT2*	2.68×10^{-2}	1.76×10^{-5}	/	/	/	/	/
Tea	*ABCG2*	7.65×10^{-3}	8.79×10^{-3}	/	/	/	/	/
Sweet beverages								
Total	*FLOT2*	5.04×10^{-3}	4.04×10^{-7}	/	/	/	/	/
Artificially sweetened beverages	*ACTR1B*	9.62×10^{-3}	1.59×10^{-2}	/	/	1.91×10^{-02}	/	/

Note: PWAS, proteome-wide association study; TWAS, transcriptome-wide association study. ROS/MAP and Banner means human brain proteomes for PWAS analysis; Frontal Cortex, Amygdala, Cerebellum, Anterior cingulate cortex and Hippocampus means five datasets of human brain gene expressions for TWAS analysis.
